# Antagonistic responses of native *Trichoderma* spp. vary among *Bipolaris sorokiniana* isolates

**DOI:** 10.1007/s00203-026-05225-6

**Published:** 2026-09-26

**Authors:** Gabriela Pupo Hagemeyer, Simone Cristine Izidoro-Morimitsu, Cacilda Marcia Duarte Rios Faria, Jackson Kawakami, Paulo Roberto Da-Silva, Felipe Liss Zchonski, Adriana Knob

**Affiliations:** 1 Department of Biological Sciences, Midwestern Parana State University, Alameda Élio Antonio Dalla Vecchia, 838, Guarapuava, PR CEP 85040-167 Brazil; 2 Department of Agronomy, Midwestern Parana State University, Alameda Élio Antonio Dalla Vecchia, 838, Guarapuava, PR CEP 85040-167 Brazil

**Keywords:** Biocontrol, Spot blotch, ISSR markers, Intraspecific variability, Fungal interactions, Araucaria forest

## Abstract

Spot blotch caused by *Bipolaris sorokiniana* is a major threat to barley production worldwide. Although the genus *Trichoderma* is widely recognized for its biocontrol potential, the extent to which genetic diversity among distinct pathogen isolates influences antagonistic responses remains poorly understood. This study screened eight native *Trichoderma* isolates from threatened Araucaria Forest remnants against six *B. sorokiniana* field isolates using exclusively in vitro assays. Pathogen genetic diversity was assessed using inter-simple sequence repeat (ISSR) markers, while biocontrol potential was evaluated through dual culture and volatile/non-volatile metabolite production. Nei’s genetic diversity (*h* = 0.444) and Shannon’s information index (*I* = 0.634) revealed high genetic diversity among *B. sorokiniana* isolates, with no obvious relationship to sampling location. Our results showed that the antagonistic efficacy of *Trichoderma* varied according to the *B. sorokiniana* isolate. Although all *Trichoderma* isolates inhibited pathogen growth, their performance was highly isolate-dependent: the commercial standard *T. harzianum* exhibited the highest inhibition (up to 94%), followed by the native isolates *T. harzianum* LMA A14 and *T. azevedoi* LMA A22 (up to 82%) in dual culture, whereas *T. asperelloides* (LMA A20 and LMA A24) and *T. longibrachiatum* LMA A21 excelled differentially in volatile and non-volatile metabolite production, showing distinct suppression profiles across different *B. sorokiniana* genotypes. This study suggests that microbial biocontrol is a dynamic interaction in which variability among pathogen isolates may influence antagonist performance, highlighting the importance of evaluating a range of them when screening broad-spectrum native biocontrol candidates for sustainable disease management.

**Supplementary Information:**

The online version contains supplementary material available at https://doi.org/10.1007/s00203-026-05225-6.

## Introduction

*Bipolaris sorokiniana* is a necrotrophic pathogen and the causal agent of spot blotch in barley, a polycyclic disease characterized by rapid conidial production and dissemination via wind, water, and seeds (Basak et al. 2024; Gupta et al. 2018). The infection process involves the release of toxins that induce severe necrosis and chlorosis, potentially leading to premature defoliation and reduced grain yield (Al-Sadi 2016). The ability of the pathogen to persist in crop residues and soil organic matter facilitates survival between growing seasons (Bessadat et al. 2023). Although conventional control relies on triazoles, strobilurins, and carboxamides, the intensive use of these agrochemicals has selected resistant isolates, underscoring the need for sustainable management alternatives (Leng et al. 2020).

Addressing these challenges requires an integrated disease management approach, combining genetic resistance, crop rotation, and cultural practices with chemical and biological control (Singh and Mehta 2019a). Biological control, which reduces pathogen inoculum or activity through antagonistic organisms (Stenberg et al. 2021), has emerged as a key strategy. These microorganisms suppress pathogens through rapid substrate colonization and other diverse antagonistic mechanisms, effectively inhibiting fungal development (Guzmán-Guzmán et al. 2023).

The filamentous fungal genus *Trichoderma* has been extensively used as a biological control agent. Its effectiveness is attributed to the broad array of secondary metabolites and hydrolytic enzymes involved in communication, natural defense, and environmental competition. The beneficial traits of *Trichoderma* have led to its use as a biofungicide, biopesticide, biofertilizer, biostimulant, and resistance inducer (Saldaña-Mendoza et al. 2023). Differences among *Trichoderma* isolates result in variable antagonistic efficiency and environmental adaptability, making the screening of local isolates better adapted to local ecological and climatic conditions highly recommended (Moya et al. 2020).

Previous studies have demonstrated the potential of *Trichoderma* spp. to control *B. sorokiniana* isolates from wheat and barley in vitro (Villa-Rodríguez et al. 2019; Shpanev and Denisyuk 2020; Momtaz et al. 2022; Metwally et al. 2024), as foliar biofungicides (Kumar et al. 2019), and as resistance inducers in wheat plants challenged with the pathogen (Singh and Malviya 2019b; Tiwari et al. 2022). However, most of these studies have evaluated antagonism against a single pathogen isolate, leaving insufficiently explored the extent to which antagonistic responses may vary among *B. sorokiniana* isolates. Because microbial interactions are governed by a dynamic, bi-directional cross-talk, distinct pathogen isolates may possess different physiological sensitivities, cell wall structures, or detoxification mechanisms, potentially influencing antagonist performance and the relative contribution of different antagonistic mechanisms. Therefore, evaluating antagonistic activity across a diverse set of pathogen isolates is essential for identifying resilient and broad-spectrum antagonists.

The Araucaria Forest, a distinct ecosystem within the Atlantic Forest biome, is recognized as a global biodiversity hotspot (Myers et al. 2000). Despite its ecological importance, this forest is currently one of the most threatened in Brazil, with only 4.3% of its original cover remaining, and just 13.5% of these remnants located within protected areas (Zorek et al. 2024), highlighting the vulnerability of this landscape.

Nonetheless, the microbial diversity harboring within these Araucaria Forest remnants remains largely underexplored, representing a valuable and untapped source of microorganisms with biotechnological potential.

Considering this vast biological heritage, we hypothesized that native *Trichoderma* isolates from Araucaria Forest remnants possess diverse antagonistic mechanisms and that their biocontrol efficacy may vary among different *B. sorokiniana* isolates. Therefore, this study aimed to evaluate the in vitro antagonistic potential of eight native *Trichoderma* isolates against six *B. sorokiniana* field isolates. Using inter-simple sequence repeat (ISSR) markers to characterize the genetic diversity of the pathogen, together with dual culture and volatile and non-volatile metabolite assays, we evaluated the variation in antagonistic responses among genetically diverse pathogen isolates.

## Materials and methods

Eight *Trichoderma* isolates previously recovered from soil samples collected in Araucaria Forest remnants in the Guarapuava region, Paraná, Brazil, were evaluated for their antagonistic potential against *B. sorokiniana*. Their molecular identities had been established in a previous barcoding study using the internal transcribed spacer (ITS) and translation elongation factor 1-alpha (*tef1*-α), complemented by RNA polymerase II second largest subunit (*rpb2*) sequences when available (Selestrino 2024). Based on the available multilocus sequence data, the isolates were identified as *T. harzianum* (LMA A14 and LMA A19), *T. asperelloides* (LMA A20 and LMA A24), *T. longibrachiatum* (LMA A21), *T. azevedoi* (LMA A22), *T. lentiforme* (LMA A26), and *T. virens* (LMA A27). The resulting sequences were deposited in the National Center for Biotechnology Information (NCBI) GenBank database and the accession numbers for the ITS, *tef1*, and *rpb2* sequences of each *Trichoderma* isolate are provided in Table S1 (Supplementary Material). Cultures were maintained in the laboratory through periodic subculturing on potato dextrose agar (PDA) and preserved using the Castellani method (Castellani 1939).

### Isolation, cultivation, and maintenance, and pathogenicity of *B. sorokiniana*

Isolates of *B. sorokiniana* (BS1–BS6) were obtained from leaf fragments of barley plants showing typical spot blotch symptoms, collected in rural areas of the municipalities of Guarapuava (25°23′36″ S, 51°27′19″ W), and Ponta Grossa (25°05′42″ S, 50°09′43″ W), Paraná, Brazil. Fragments were surface disinfected in 0.5% sodium hypochlorite (NaClO) for 30 s, rinsed in sterile distilled water, dried on sterile paper towels, and placed onto PDA plates. Cultures were incubated in the dark for 4 days at 25 ± 2 °C. Successive subcultures were performed until pure cultures were obtained. All isolates were preserved using the Castellani method (Castellani 1939).

The pathogenicity of all *B. sorokiniana* isolates was demonstrated on barley seedlings prior to the in vitro experiments. For this purpose, barley plants (*Hordeum vulgare* L. cv. Danielle, susceptible to the pathogen) were grown. Seeds were initially disinfected in 70% ethanol for 1 min, rinsed three times with sterile distilled water, immersed in 1% sodium hypochlorite for 10 min, and rinsed ten times with sterile water. The disinfected seeds were then dried between sterile filter papers for 24 h. Sowing was performed in 5-L pots filled with a mixture of soil and sand (2:1 ratio), which had been fertilized with NPK 13-15-12 (2.5 g pot) and homogenized 10 days prior to planting. Six seeds per pot were sown at a depth of 2 cm.

For inoculum preparation, the pathogen was cultured on PDA-V8 medium (200 mL V8 juice, 10 g PDA, 10 g agar, 3 g CaCO_3_ and 800 mL distilled water) using a specific thermal and light cycle to induce sporulation: 5 days in the dark at room temperature (20–25 °C), 7 days under a 12-h photoperiod at 25 °C, and finally 3 days in the dark at 15–20 °C. Conidia were dislodged by adding sterile distilled water to the plates and using a Drigalsky loop; this process was performed twice to optimize spore release. The resulting solution was filtered through glass wool, and the concentration was adjusted to 9.0 × 10^6^ conidia mL^− 1^ using a Neubauer chamber.

Plants were spray-inoculated until runoff at the second-leaf stage (Large, 1954) using manual spray bottles. Control plants were sprayed with sterile distilled water only. Immediately after inoculation, the pots were covered with black polypropylene plastic bags for 24 h to create a humid chamber (100% RH) to facilitate pathogen penetration. The pots were maintained in a greenhouse for 46 days, with temperatures ranging from 2 to 35 °C. Visual assessments of disease incidence and severity were conducted every five days, starting five days post-inoculation, completing a total of six evaluations. The typical symptoms of spot blotch were successfully observed and recorded on all inoculated leaves, confirming the pathogenicity of the target isolates, whereas control plants remained completely asymptomatic.

### Morphological identification and characterization of *B. sorokiniana*

Species identification was performed using a polyphasic approach based on cultural, morphological, and molecular characteristics. For morphological characterization, pure cultures were grown on potato dextrose agar (PDA) at 25 ± 2 °C for 7 days. Colony characteristics, including color, texture, pigmentation, exudate production, margin characteristics, and growth pattern, were recorded according to Gupta et al. (2018), Debnath et al. (2022), Sharmin et al. (2022), and Yadav et al. (2022). Microscopic characteristics, including conidial shape and pigmentation were qualitatively examined under a light microscope.

### Molecular identification of *B. sorokiniana*

Pathogen isolates were grown on PDA for 7 days at 25 ± 2 °C. Harvested mycelia were ground to a fine powder in liquid nitrogen and immediately transferred into sterile 1.5 mL microtubes. Total genomic DNA was then extracted following the cetyltrimethylammonium bromide (CTAB)-based method of Doyle and Doyle (1987). DNA quality was assessed on 1% agarose gels, and DNA concentration was estimated using a NanoDrop 2000/2000c spectrophotometer (Thermo Scientific, CA, USA).

Identification of *B. sorokiniana* isolates was based on sequencing of the ITS region, using primers ITS4 (5’-TCCTCCGCTTATTGATATGC-3’) and ITS5 (5’-GGAAGTAAAAGTCGTAACAAGG-3’) (White et al. 1990). Amplification reactions were performed in a Veriti thermocycler (Applied Biosystems) in a volume of 50 µL using 25 ng of DNA, 1× polymerase chain reaction (PCR) buffer with 1.0 mM MgCl₂, 0.25 mM of each deoxynucleoside triphosphate (dNTP), 1 U Taq DNA polymerase, 0.4 µM of each primer (final concentration), and ultrapure water. The PCR program consisted of initial DNA denaturation at 95 °C for 3 min, followed by 35 cycles of DNA denaturation at 95 °C for 15 s, primer annealing at 53 °C for 15 s, and extension at 72 °C for 1 min, with a final extension at 72 °C for 10 min.

Amplification of the expected 600–700 bp (bp) fragment was confirmed by electrophoresis using 10 µL of the PCR product on a 1.8% agarose gel stained with ethidium bromide (0.5 µg mL⁻¹). Following confirmation of amplification, the remaining 40 µL of the PCR products were purified with absolute ethanol, quantified using NanoDrop 2000/2000c spectrophotometer (Thermo Scientific, CA, USA). The purified product was sent to the company ACTGene Análises Moleculares LTDA (Alvorada, RS, Brazil) for sequencing by Sanger method on an ABI 3500 Genetic Analyzer (Applied Biosystems). The sequences were inspected for quality using BioEdit v.7.2.5 (Hall 1999). Homology searches were performed using Basic Local Alignment Search Tool for nucleotides (BLASTn) (Altschul et al. 1997) in the NCBI database (Sayers et al. 2025). To verify the species identification obtained via BLAST, ITS sequences from 22 *Bipolaris* species were retrieved from GenBank and used to construct a phylogenetic tree using the maximum likelihood method with IQ-TREE software (Nguyen et al. 2015). The ITS sequences were deposited in GenBank under the accession numbers PX735807–PX735812. Species identification was supported by the combination of morphological characteristics, pathogenicity, and ITS sequence similarity by BLAST and phylogenetic analysis.

### ISSR marker analysis

To perform the genetic similarity analysis between *B. sorokiniana* isolates, the Inter-Simple Sequence Repeat (ISSR) technique was used. Genomic DNA from each isolate was extracted as described in the molecular identification step and amplified using a set of 10 ISSR primers developed by the University of British Columbia (UBC), Vancouver, Canada (Table 1). The ISSR primers were initially selected based on their reported performance in previous studies, considering the number of amplified bands, percentage of polymorphism, polymorphism information content (PIC), and resolution power reported by Fedrigo et al. (2016), as well as the number of amplified bands and percentage of polymorphism reported by de Arruda et al. (2021). Although these studies evaluated the primers in other fungal species, they were used as a basis for the initial selection of candidate primers. Their applicability to *B. sorokiniana* was subsequently empirically evaluated in the present study, and primers producing clear and reproducible amplification profiles were retained for the genetic analysis.

**Table 1:** List of the 10 ISSR (inter-simple sequence repeat) primers used to assess the genetic variability of *B. sorokiniana* isolates

Primer	Sequence*	T_a_ (°C)	NAF	*P* (%)
UBC 807	(AG)_8_T	52	10	0.00
**UBC 808**	(AG)_8_C	50	16	81.25
**UBC 809**	(AT)_8_T	55	15	93.33
**UBC 811**	(GA)_8_C	53	12	66.66
**UBC 827**	(AC)_8_G	53	10	10.00
**UBC 835**	(AG)_8_YC	54	9	55.55
UBC 848	(CA)_8_RG	55	9	0.00
UBC 861	(ACC)_6_	52	0	0.00
**UBC 868**	(GAA)_6_	50	11	54.54
**UBC 873**	(GACA)_4_	50	8	12.50

The annealing temperature (*T*_a_, °C), total number of amplified fragments (NAF), and percentage of polymorphism (P) for each primer are provided. Primers in bold were those used in the analyses

*Y = (C, T); R = (A, G). Primers UBC 807 and UBC 848 were monomorphic (*P* = 0.00), and primer UBC 861 failed to amplify (NAF = 0); all three were discarded from the subsequent analysis

PCR was performed in a final volume of 12.5 µL containing approximately 20 ng of genomic DNA, 0.2 µM of primer, 200 µM of each dNTP, 1.5 mM MgCl₂, 1 U Taq DNA polymerase, and 1× reaction buffer [200 mM Tris-HCl (pH 8.4), 500 mM KCl]. Amplifications were carried out in a Veriti thermocycler (Applied Biosystems) using the following cycling conditions: an initial denaturation at 94 °C for 5 min, followed by 35 cycles of denaturation at 94 °C for 45 s, primer-specific annealing temperature (Table 1) for 30 s, and extension at 72 °C for 1 min. A final step at 72 °C for 10 min was added to complete fragment extension.

PCR products were run on 1.8% agarose gels stained with ethidium bromide (0.5 µg mL⁻¹) and electrophoresed for approximately 4 h at 110 V. Fragment sizes were estimated using a 100-bp DNA ladder (Invitrogen). Bands were visualized under UV light and photodocumented using the L-PIX EX digital imaging system (Loccus do Brasil).

Of the 10 primers initially used, seven that provided clear, intense, and distinct DNA bands were selected for the final analysis, while primers that failed to amplify or produced unscoreable profiles or were monomorphic were completely discarded. To ensure high reproducibility and reliability in the genetic analysis, only clear and reproducible DNA bands were scored. The binary matrix (band present = 1, band absent = 0) obtained from the amplification data of all primers was used in subsequent analyses. Genetic similarity among isolates was estimated using the Jaccard coefficient (Sneath and Sokal 1973) and the dendrogram was constructed using the unweighted pair group method with arithmetic mean (UPGMA) using NTSYSpc software version 2.1 (Rohlf 2000). Node support was evaluated by bootstrap analysis with 1,000 replicates also using NTSYSpc software. The genetic groups were defined based on the average similarity among all isolates, obtained using the pairwise similarity matrix generated during the construction of the dendrogram. PopGene version 1.32 was used to calculate descriptive parameters, including Nei’s gene diversity (*h*), Shannon’s Information index (*I*), the number of polymorphic loci, and the percentage of polymorphism, while monomorphic loci were excluded from these analyses. Genetic diversity values are presented as means ± standard deviation. The genetic groups were defined based on the average similarity among all isolates, obtained using the pairwise similarity matrix generated during the construction of the dendrogram.

### Assessment of *B. sorokiniana* antagonism by *Trichoderma* isolates in dual culture

Antagonistic activity in dual culture was evaluated following Dennis and Webster (1971). PDA plates (90 mm) were inoculated with 5-mm mycelial discs obtained from seven-day-old cultures of each *Trichoderma* isolate and *B. sorokiniana.* A commercial wettable powder formulation of *Trichoderma harzianum* (Ecotrich^®^ WP, Ballagro Agro Tecnologia Ltda., Bom Jesus dos Perdões, SP, Brazil; strain ESALQ-1306, containing a minimum of 2 × 10^9^ viable conidia g^-1^ was used as a standard reference treatment. The powder was spread onto PDA plates using a sterile inoculation loop, incubated for 7 days, and used to obtain active mycelial discs. The native *Trichoderma* isolates were likewise maintained and successively subcultured on PDA under laboratory conditions before the assays, and mycelial discs were obtained from actively growing colonies. Thus, both the commercial strain and the native isolates were subjected to standardized culture conditions prior to their use in the antagonism assays.

The discs were placed on opposite sides of the plate, 7 cm apart, and incubated at 25 ± 2 °C for 7 days. For the control treatments, the pathogen disc was placed in the exact same eccentric position (near the edge of the plate) to ensure standard spatial conditions. After incubation, the pathogen colony diameter was measured along a single axis passing through the center of the colony, oriented parallel to the confrontation line, and the inhibition rate (%) was calculated according to Royse and Ries (1978):

$$ Inhibition~rate~(\% )~ = ~[(R1~ - ~R2)~/~R1]~ \times ~100 $$

where R1 is the colony diameter of the control (pathogen alone), and R2 is the diameter in the presence of the antagonist.

### Assessment of the inhibition effect of volatile secondary metabolites (VOCs) from *Trichoderma* isolates against *B. sorokiniana*

Volatile-mediated inhibition was evaluated according to Dennis and Webster (1971). PDA plates were inoculated with 5-mm mycelial discs of each *Trichoderma* isolate or *B. sorokiniana* and incubated for 24 h at 25 ± 2 °C to allow initial establishment. For the control treatment, a PDA plate inoculated with *B. sorokiniana* was paired with a plate containing fresh, uninoculated PDA medium. For VOC assessment, the lids were removed, and the plate containing the pathogen was inverted over the base containing the antagonist. The paired plates were sealed together with Parafilm^®^ and incubated at 25 ± 2 °C for 7 days. After incubation, the pathogen colony diameter was measured, and the inhibition percentage was calculated as described above.

### Assessment of the inhibition effect of non-volatile secondary metabolites (non-VOCs) from *Trichoderma* isolates against *B. sorokiniana*

The effect of non-volatile metabolites was assessed following Dennis and Webster (1971). Each *Trichoderma* isolate was grown in 250 mL Erlenmeyer flasks containing 125 mL of potato dextrose broth (PDB) and inoculated with five 5-mm mycelial discs from 7-day-old cultures. Flasks were incubated at 150 rpm and 25 ± 2 °C for 5 days.

Cell-free culture filtrates were obtained by vacuum filtered through filter paper and subsequently through sterile cellulose membranes (0.22 μm) and used immediately after filtration. Aliquots of the cell-free culture filtrate were plated onto PDA and monitored for 48 h to confirm the total absence of microbial growth before experimental use. The sterile filtrates were incorporated into PDA at 25% (v/v) by mixing with molten PDA cooled to approximately 45 °C. Control plates consisted of PDA medium mixed with sterile uninoculated broth instead of the fungal filtrate. Plates were then inoculated with a single 5-mm disc of *B. sorokiniana* and incubated at 25 ± 2 °C for 7 days. After incubation, colony diameters of *B. sorokiniana* were measured, and inhibition was quantified as previously described.

### Statistical analyses

Each treatment consisted of three experimental replicates (one Petri dish per replicate), and the entire experiment was conducted twice independently. To confirm that pooling of data from the two independent experimental runs was appropriate, a two-way ANOVA was performed for each assay, considering *Trichoderma* isolate and experimental run as factors. No significant main effect of experimental rum (*p* > 0.05) nor a significant treatment x run interaction (*p* > 0.05) was observed for any of the assays, supporting the pooling of data from both independent runs for the subsequent analyses. Accordingly, corresponding replicates from the two experimental runs were averaged, resulting in three pooled observations per treatment combination. Thus, although six raw observations were obtained for each treatment combination (three replicates in each of two independent runs), the subsequent factorial ANOVA was performed using three pooled observations per treatment combination. The means presented in the tables were calculated from these three pooled observations.

Prior to the analysis of variance (ANOVA), the assumptions of normality of residuals and homogeneity of variances were checked and confirmed using Shapiro–Wilk and Levene’s tests, respectively. To evaluate the interactions and compare the means among the different *Trichoderma* isolates and *B. sorokiniana* strains, Tukey’s test was applied using SISVAR software version 5.6 (Ferreira 2019). Additionally, to determine whether each *Trichoderma* treatment significantly reduced pathogen growth compared to the pathogen-only control, mean comparisons were performed using Dunnett’s many-to-one test via the multcomp package in R software (version 4.6.1).

## Results

### Morphological, molecular and pathogenic characterization of *B. sorokiniana*

**Fig. 1:**
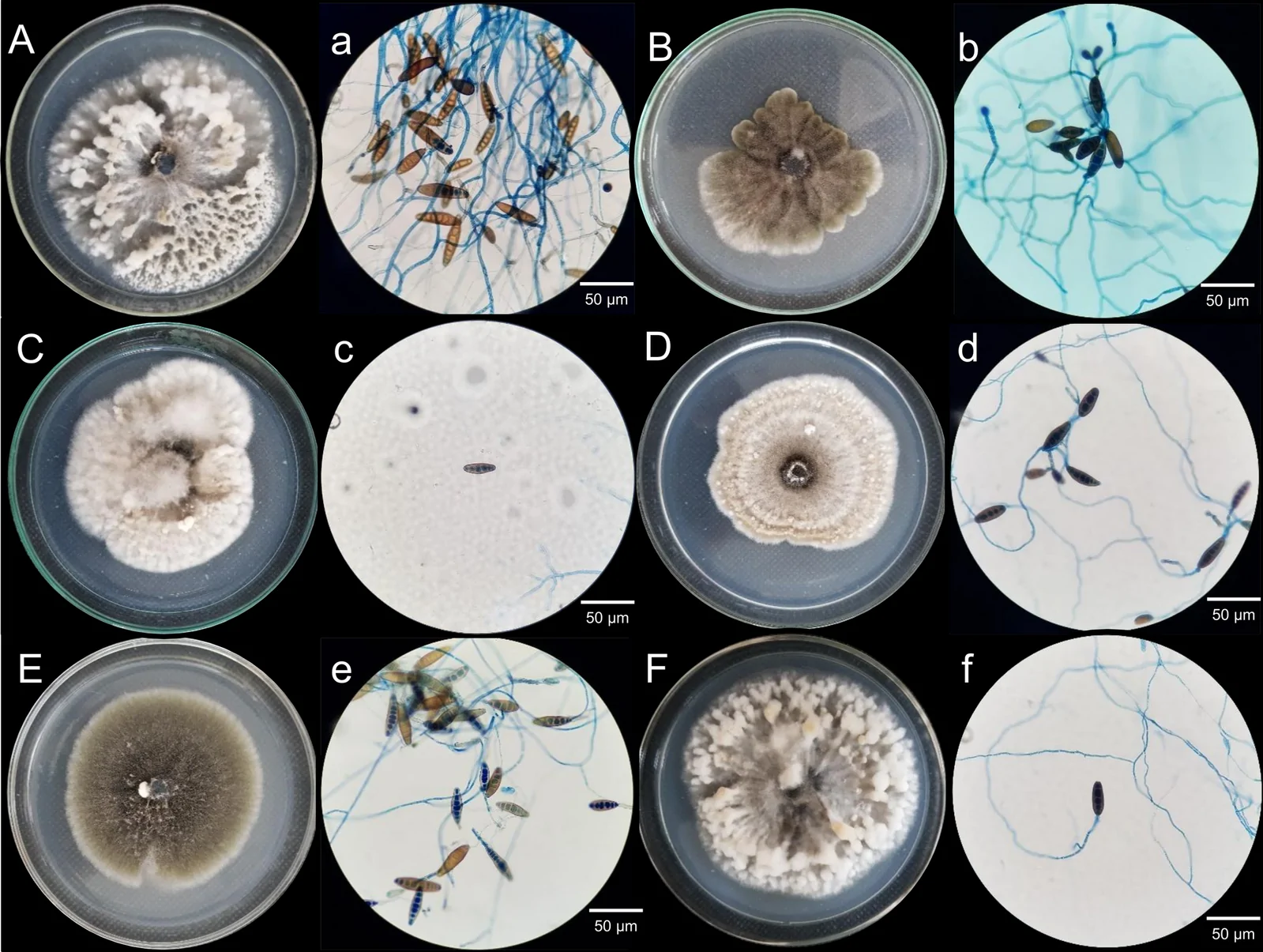
Macroscopic (**A**-**F**) and microscopic (**a**-**f**, 400×) morphology of six *B. sorokiniana* isolates. Microscopic structures were obtained using the slide micro-culture technique and incubated at 25 °C for 4 days. **A**, **a**: BS1; **B**, **b**: BS2; **C**, **c**: BS3; **D**, **d**: BS4; **E**, **e**: BS5; **F**, **f**: BS6

Six isolates of *B. sorokiniana* (BS1–BS6) were recovered from barley leaves showing typical brown spot lesions. Isolates BS1, BS3, and BS6 originated from barley crops in the Guarapuava/PR region, whereas the remaining isolates came from Ponta Grossa/PR. Morphological characterization was based on colony (macroscopic) and microscopic features. The isolates exhibited marked phenotypic variability (Fig. 1). On PDA, colony coloration ranged from cottony white to dark melanized. BS1 and BS6 developed dark gray mycelia, whitish aerial tufts and circular margins (Fig. 1A and F, respectively).

BS2 showed brownish-gray, cottony colonies with irregular margins (Fig. 1B). BS3 displayed white-brown cottony mycelium with irregular margins (Fig. 1C), whereas BS4 formed velvety white-brown colonies with a darkened center (Fig. 1D). BS5 exhibited grayish, cottony mycelia with light, circular borders (Fig. 1E).

Micromorphological observations revealed simple, rarely branched, septate conidiophores. Conidia were produced singly at the apex of conidiogenous cells and were light brown to golden, curved, with multiple transverse septa and tapered apices, morphological features characteristic of *B. sorokiniana*. Molecular identification was confirmed through ITS-rDNA sequencing. The obtained sequences showed > 99.82% similarity to *B. sorokiniana* reference sequences deposited in public databases, confirming the identity of all isolates. Furthermore, the phylogenetic tree clearly evidenced the clustering of all obtained sequences within the *B. sorokiniana* clade, confirming the identity of all isolates (Figure S1).

The pathogenicity of the target isolates was demonstrated on barley seedlings prior to the bioassays, as all inoculated plants developed typical spot blotch symptoms, while control plants remained asymptomatic. Taken together, the macro- and micromorphological analyses, combined with the molecular identification, phylogenetic tree clustering, and pathogenicity assays confirmed the identity of all six isolates as *B. sorokiniana*.

### Genetic diversity of *B. sorokiniana* based on ISSR markers

Of the 10 ISSR primers evaluated, seven exhibited clear banding profiles and polymorphism. These seven primers yielded a total of 81 amplified bands, of which 48 were polymorphic (Table 1). Among these primers, polymorphism rates varied substantially, ranging from 10.00% (primer UBC 827) to 93.33% (primer UBC 809). The overall percentage of polymorphic loci was 59.26%. Overall, the ISSR marker profiles revealed a high degree of genetic diversity among the investigated *B. sorokiniana* isolates. The mean Nei’s gene diversity (*h*) was 0.4444 (± 0.0743). Additionally, Shannon’s Information Index (*I*) averaged 0.6342 (± 0.0811), indicating substantial genetic variation among the analyzed isolates. The average Jaccard genetic similarity among the isolates was 0.30, indicating high genetic diversity within the investigated set of isolates. Based on the UPGMA dendrogram, a similarity threshold of 0.33 was used to delimit the major genetic clusters.

**Fig. 2:**
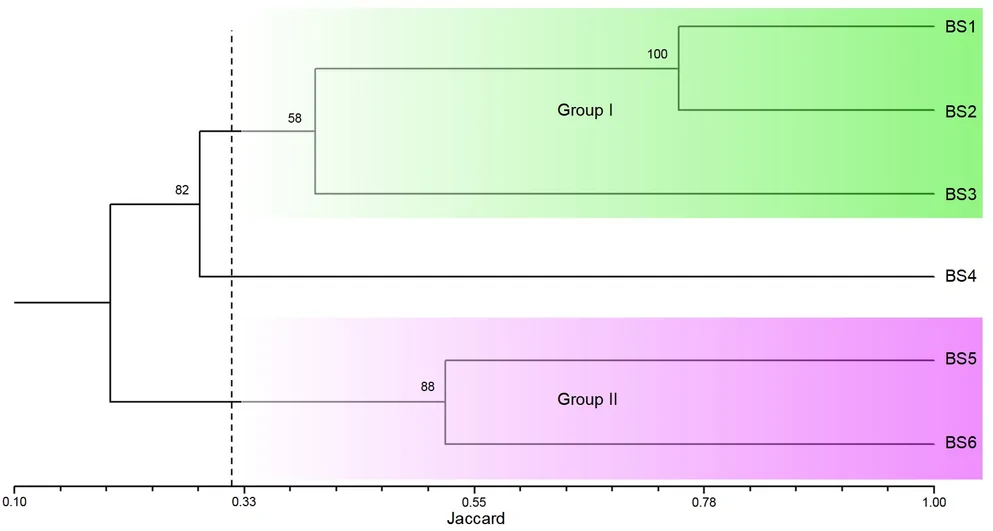
Dendrogram showing the genetic relationships among *B. sorokiniana* isolates based on ISSR markers. The analysis was performed using the Jaccard similarity coefficient and the UPGMA clustering method. Numbers at the nodes represent bootstrap support values based on 1000 replicates. The dashed line indicates the similarity threshold (0.33) used to define major clusters

The UPGMA dendrogram, supported by 1,000 bootstrap replicates, demonstrated high statistical confidence for three of the four nodes (Fig. 2). The first cluster comprised isolates BS1, BS2, and BS3; within this group, BS1 and BS2 showed the highest similarity, indicating their close genetic relationship.

The second cluster included isolates BS5 and BS6, which formed a distinct group separated from the remaining genotypes. In contrast, isolate BS4 formed an independent branch and did not cluster with the remaining isolates at the adopted similarity threshold, indicating marked genetic divergence.

Regarding geographic origin, isolates BS1, BS3, and BS6 were obtained from Guarapuava, whereas BS2, BS4, and BS5 originated from Ponta Grossa, municipalities located approximately 160 km apart. No clear association between genetic clustering and geographic origin was observed within this sample set, as isolates from both municipalities occurred within the same genetic groups.

### Antagonism in dual culture

Eight *Trichoderma* isolates were evaluated for their biocontrol activity against six isolates of *B. sorokiniana.* ANOVA revealed significant effects for the *Trichoderma* isolate effect (F = 31.14, *p* < 0.01), the pathogen isolate effect (F = 3.07, *p* < 0.01), and their interaction (F = 3.23, *p* < 0.01) (Table 2).

**Table 2:** In vitro mycelial growth inhibition of *B. sorokiniana* by *Trichoderma* isolates in dual culture assays after seven days at 25 ± 2 °C

*Trichoderma* isolates	Mycelial growth inhibition (%)					
	*B. sorokiniana* isolates					
	BS1	BS2	BS3	BS4	BS5	BS6
*T. harzianum* LMA A14	78.05 abA	82.51 abA	73.65 bcAB	75.96 abAB	65.71 bcB	72.82 abAB
*T. harzianum* LMA A19	79.56 aA	71.52 bcdAB	71.60 bcAB	69.70 bAB	74.74 abAB	67.48 bB
*T. asperelloides* LMA A20	75.16 abAB	65.97 dB	67.82 cdB	70.54 abAB	72.31 abAB	79.88 aA
*T. longibrachiatum* LMA A21	66.48 bcA	62.74 dA	51.92 eB	57.95 cAB	57.37 cAB	65.15 bA
*T. azevedoi* LMA A22	80.87 aA	73.68 bcdA	76.40 bcA	75.36 abA	78.14 aA	74.36 abA
*T. asperelloides* LMA A24	77.48 abAB	79.12 bcAB	82.25 bA	69.40 bcB	72.75 abAB	72.64 abAB
*T. lentiforme* LMA A26	61.00 cB	62.57 dB	58.33 deB	67.47 bcAB	73.72 abA	66.26 bAB
*T. virens* LMA A27	61.25 cAB	68.42 cdAB	57.88 deB	68.92 bcA	63.20 bcAB	65.28 bAB
Standard (*T. harzianum)*	85.66 aAB	91.35 aAB	94.49 aA	81.93 aB	83.33 aB	82.03 aB

Inhibition values were calculated using the percentage inhibition of mycelial growth

ANOVA summary: *Trichoderma* factor (F (8,108) = 31.14, *p* < 0.01), pathogen factor (F (5, 108) = 3.07, *p* < 0.01) and their interaction (F (40, 108) = 3.23, *p* < 0.01). Same uppercase letter in rows (*B. sorokiniana* isolates) and same lowercase letter in columns (*Trichoderma* isolates) do not differ by Tukey’s test (*p* < 0.05). Values represent the mean of three pooled observations (*N* = 3), with each pooled observation calculated as the mean of the corresponding replicate from the two independent experimental runs

**Fig. 3:**
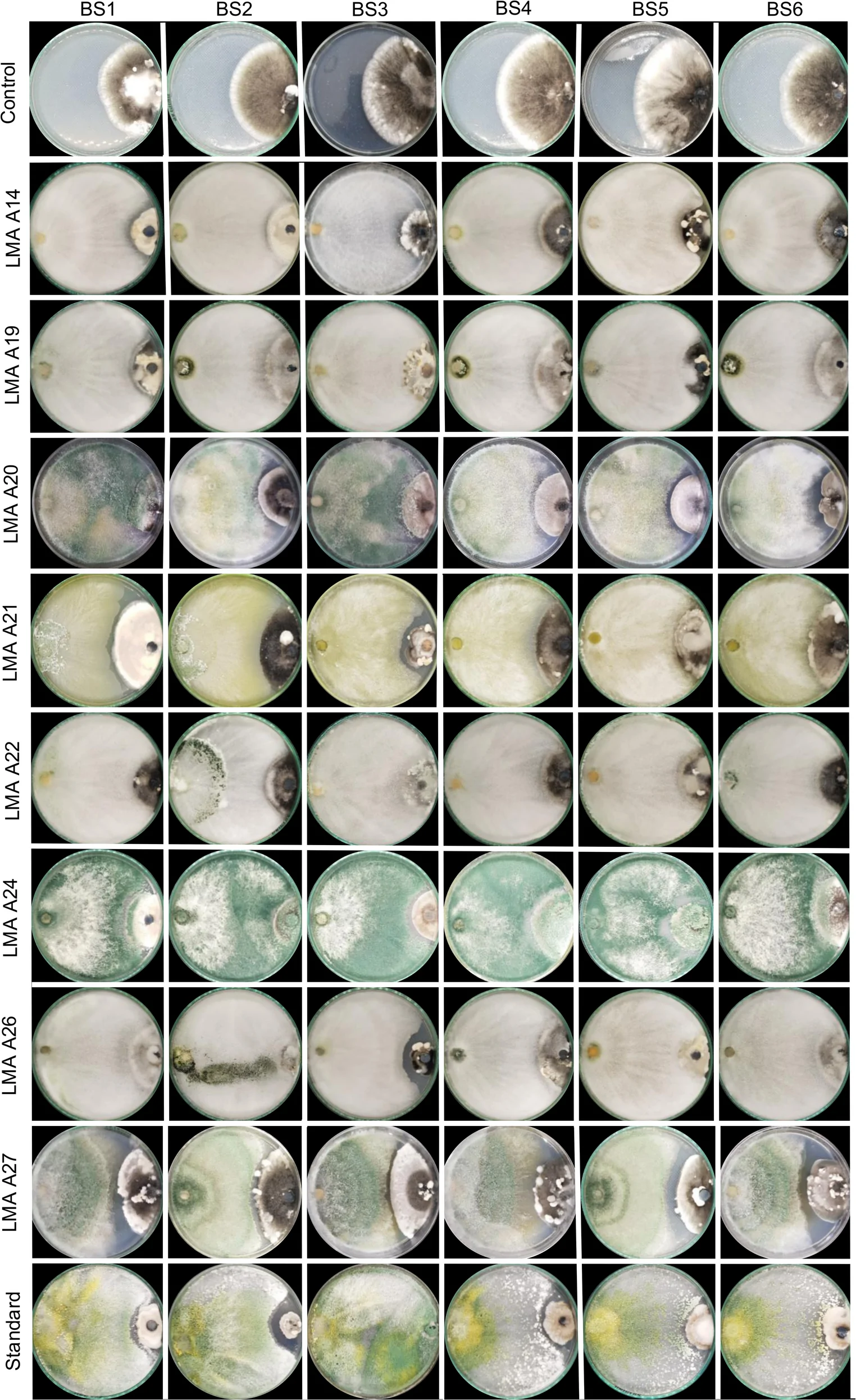
Dual culture assays showing the antagonistic activity of *Trichoderma* isolates against *B. sorokiniana*. Plates were incubated at 25 ± 2 °C for 7 days. *Trichoderma* isolates rapidly colonized the medium, suppressing pathogen growth through overgrowth or formation of inhibition zones. Columns represent the *B. sorokiniana* isolates (BS1 to BS6). Rows represent the antagonist treatments (native *Trichoderma* isolates LMA A14 to LMA A27), including a control (pathogen grown alone) and a standard commercial treatment. In each panel, *Trichoderma* was inoculated on the left side and *B. sorokiniana* on the right side

All *Trichoderma* isolates inhibited the mycelial growth of *B. sorokiniana* when compared with the control (Dunnett’s test, *p* < 0.001) (Table S2), with inhibition ranging from 51.92 to 82.51% (Table 2). Among the native antagonists, *T. azevedoi* LMA A22 and *T. harzianum* LMA A14 stood out as the most effective isolates. *Trichoderma longibrachiatum* LMA A21 and *T. virens* LMA A27 displayed the lowest inhibition percentages (51.92–66.48% and 57.88–68.92%, respectively) in dual culture assays (Table 2). During incubation, *Trichoderma* isolates rapidly colonized the culture medium and frequently overgrew the pathogen colonies (Fig. 3). Physical contact generally occurred only after substantial expansion of the antagonist colony, resulting in restricted colony diameter of *B. sorokiniana*.

Visual assessment indicated that isolates such as *T. longibrachiatum* LMA A21, *T. lentiforme* LMA A26, *T. virens* LMA A27, and the commercial standard formed clear inhibition zones suggestive of antibiosis. In contrast, isolates including *T. harzianum* LMA A14 and LMA A19, *T. asperelloides* LMA A20 and LMA A24, and *T. azevedoi* LMA A22 exhibited abundant sporulation over pathogen hyphae, a patter consistent with putative mycoparasitic behavior. Regarding pathogen response, isolates BS1, BS4, BS5, and BS6 were the most susceptible.

### Volatile secondary metabolites

A significant effect of *Trichoderma* isolate (F = 31.05, *p* < 0.01), a significant effect of *B. sorokiniana* isolate (F = 73.4, *p* < 0.01), and their interaction (F = 6.64, *p* < 0.01) were observed (Table 3). Most *Trichoderma* isolates produced volatile compounds that reduced pathogen growth relative to the control (Dunnett’s test, *p* < 0.05) (Table S3), with the inhibitory effect varying depending on the specific antagonist isolate and pathogen strain. Negative inhibition values were observed in some *Trichoderma*–*B. sorokiniana* combinations in volatile metabolite assays, indicating stimulation of pathogen growth relative to the corresponding controls (Table 3).

**Table 3:** In vitro inhibition of *B. sorokiniana* mycelial growth by volatile secondary metabolites (VOCs) produced by *Trichoderma* isolates

*Trichoderma* isolates	Mycelial growth inhibition (%)					
	*B. sorokiniana* isolates					
	BS1	BS2	BS3	BS4	BS5	BS6
*T. harzianum* LMA A14	39.66 bcBC	54.63 aAB	61.87 abA	30.10 aCD	40.09 bBC	14.04 cD
*T. harzianum* LMA A19	24.78 cdB	57.37 aA	55.49 abcA	29.20 aB	56.26 abA	21.17 bcB
*T. asperelloides* LM A20	60.7 aA	40.75 abBC	67.16 aA	26.30 abC	50.42 abAB	37.08 abBC
*T. longibrachiatum* LMA A21	51.1 abAB	52.99 aAB	52.57 abcAB	28.52 abC	65.35 aA	41.47 abBC
*T. azevedoi* LMA A22	20.61 cdBC	29.79 abAB	39.22 cdAB	-14.30 cD	39.94 bA	3.30 cCD
*T. asperelloides* LMA A24	39.87 bcAB	41.08 abAB	51.24 abcA	29.20 aB	40.99 bAB	22.95 bcB
*T. lentiforme* LMA A26	-18.37 eE	27.15 bcCD	44.06 bcdBC	8.62 bDE	63.56 aA	51.93 aAB
*T. virens* LMA A27	13.98 deBC	13.79 bcBC	28.44 dB	-13.62 cC	50.82 abA	6.84 cC
Standard (*T. harzianum)*	61.41 aAB	79.58 aA	54.90 abcB	61.63 aAB	50.37 abB	55.29 aB

Plates were incubated at 25 ± 2 °C for seven days

ANOVA summary: *Trichoderma* factor (F (8, 108) = 31.05, *p* < 0.01), pathogen factor (F (5, 108) = 73.4, *p* < 0.01) and their interaction (F (40, 108) = 6.64, *p* < 0.01). Means followed by the same uppercase letter in rows (*B. sorokiniana* isolates) and same lowercase letter in columns (*Trichoderma* isolates) do not differ by Tukey’s test (*p* < 0.05). Values represent the mean of three pooled observations (*N* = 3), with each pooled observation calculated as the mean of the corresponding replicate from the two independent experimental runs. Negative values indicate a stimulation of mycelial growth compared to the control treatment

*Trichoderma asperelloides* LMA A20 and *T. longibrachiatum* LMA A21 were the most effective, showing inhibition ranges of 26.03–67.16% and 28.52–65.35%, respectively. Together with the commercial standard, these isolates significantly suppressed the mycelial growth of all six *B. sorokiniana* isolates.

**Fig. 4:**
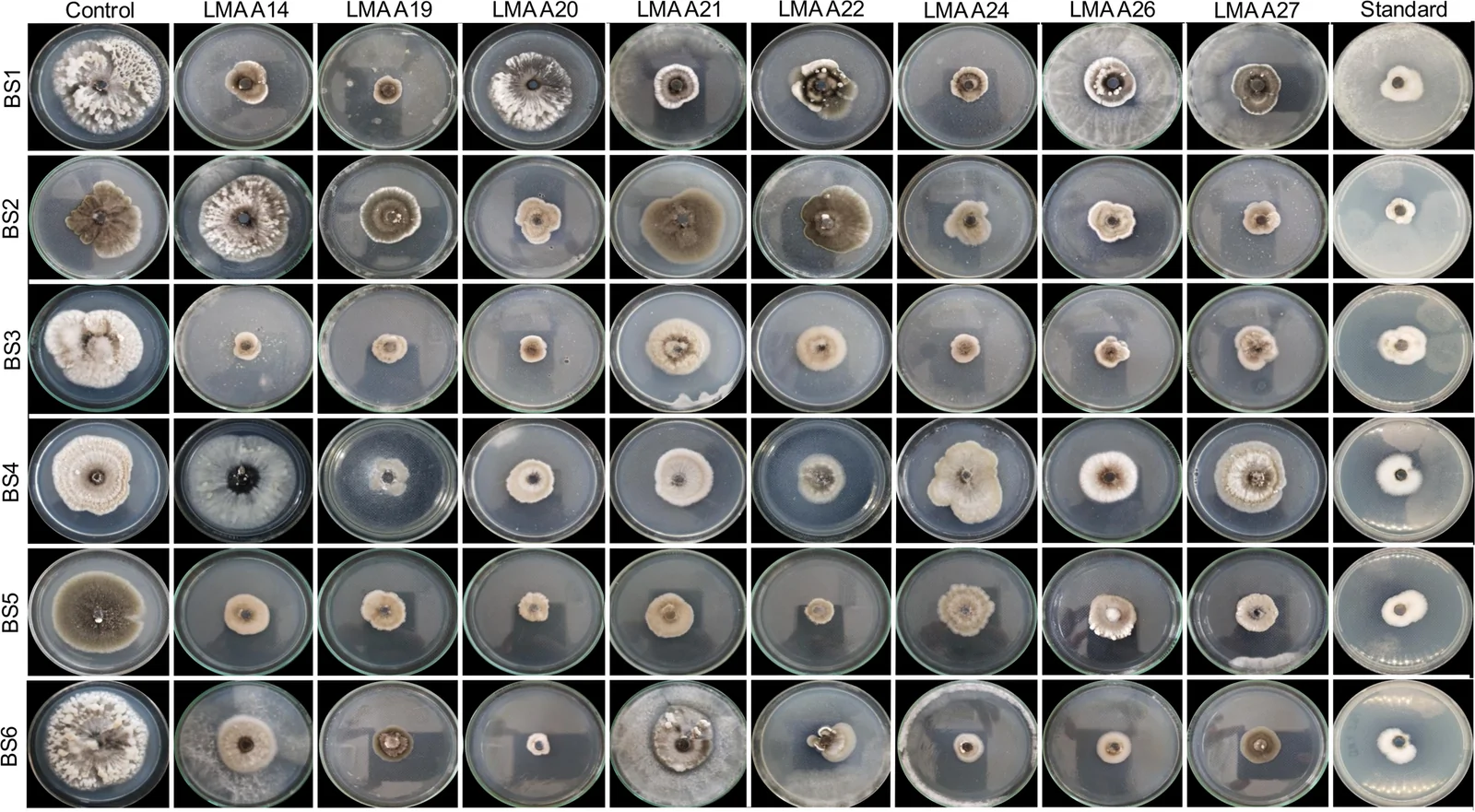
Inhibition of *B. sorokiniana* by volatile secondary metabolites (VOCs) produced by *Trichoderma* isolates. For the control treatment, a PDA plate inoculated with *B. sorokiniana* was paired with a plate containing fresh, uninoculated PDA medium. Plates were incubated at 25 ± 2 °C for seven days

Volatile metabolites from *T. harzianum* LMA A14 and LMA A19, and *T. asperelloides* LMA A24, significantly reduced the growth of three pathogen isolates. *T. lentiforme* LMA A26 inhibited two isolates, whereas *T. azevedoi* LMA A22 and *T. virens* LMA A27 suppressed one isolate each (Table 3; Fig. 4).

Isolate BS5 was the most susceptible to volatile metabolites, being suppressed by seven of the eight *Trichoderma* isolates. In contrast, BS4 was the least affected, consistently showing the lowest inhibition percentages across all antagonists tested.

### Non-volatile secondary metabolites

A significant effect of *Trichoderma* isolate (F = 118.61, *p* < 0.01), a significant effect of *B. sorokiniana* isolate (F = 50.40, *p* < 0.01) and their interaction (F = 2.89, *p* < 0.01) were observed (Table 4). Most *Trichoderma* isolates produced non-VOCs that significantly reduced pathogen growth compared with the control (Dunnett’s test, *p* < 0.05) (Table S4), although the inhibitory effect varied depending on the specific antagonist isolate and pathogen strain. Negative inhibition values were observed for some *Trichoderma–B. sorokiniana* combinations, indicating that pathogen growth was slightly higher in the presence of non-volatile metabolites than in the corresponding controls (Table 4).

**Table 4:** Effects of cell-free culture filtrates from *Trichoderma* isolates on the mycelial growth of *B. sorokiniana*

*Trichoderma* isolates	Mycelial growth inhibition (%)					
	*B. sorokiniana* isolates					
	BS1	BS2	BS3	BS4	BS5	BS6
*T. harzianum* LMA A14	36.23 bcCD	59.82 abA	55.18 abAB	38.79 bBCD	41.69 bcBC	23.24 bD
*T. harzianum* LMA A19	15.58 deB	16.08dBC	43.74 bA	27.15 bcAB	22.07 deB	-22.21 dC
*T. asperelloides* LMA A20	4.90 eB	27.42 cdA	19.66 cA	-7.09 dC	6.44 eB	-38.35 eD
*T. longibrachiatum* LMA A21	66.02 aAB	74.77 aA	68.91 aA	73.86 aA	63.4 aAB	50.17 aB
*T. azevedoi* LMA A22	33.36 cdA	33.10 cdA	23.25 cAB	15.00 bcB	24.8 cdAB	-23.99 dC
*T. asperelloides* LMA A24	29.88 cdB	24.90 cdB	47.53 bA	17.12 cdC	21.27 deB	-20.98 dD
*T. lentiforme* LMA A26	16.50 deAB	29.04 cdA	22.51 cA	15.78 cdB	16.27 deAB	-30.69 eC
*T. virens* LMA A27	53.88 abA	52.70 bA	49.53 bA	36.68 bAB	49.28 abA	21.67 bB
Standard (*T. harzianum)*	42.59 bcB	42.83 bcB	57.75 abAB	66.27 aA	43.43 bB	8.41 cC

Plates were incubated at 25 ± 2 °C for seven days

ANOVA summary: *Trichoderma* factor (F (8, 108) = 118.61, *p* < 0.01), pathogen factor (F (5, 108) = 50.4, *p* < 0.01) and their interaction (F (40, 108) = 2.89, *p* < 0.01). Means followed by the same uppercase letter in rows (*B. sorokiniana* isolates) and same lowercase letter in columns (*Trichoderma* isolates) do not differ by Tukey’s test (*p* < 0.05). Values represent the mean of three pooled observations (*N* = 3), with each pooled observation calculated as the mean of the corresponding replicate from the two independent experimental runs. Negative values indicate a stimulation of mycelial growth compared to the control treatment

**Fig. 5:**
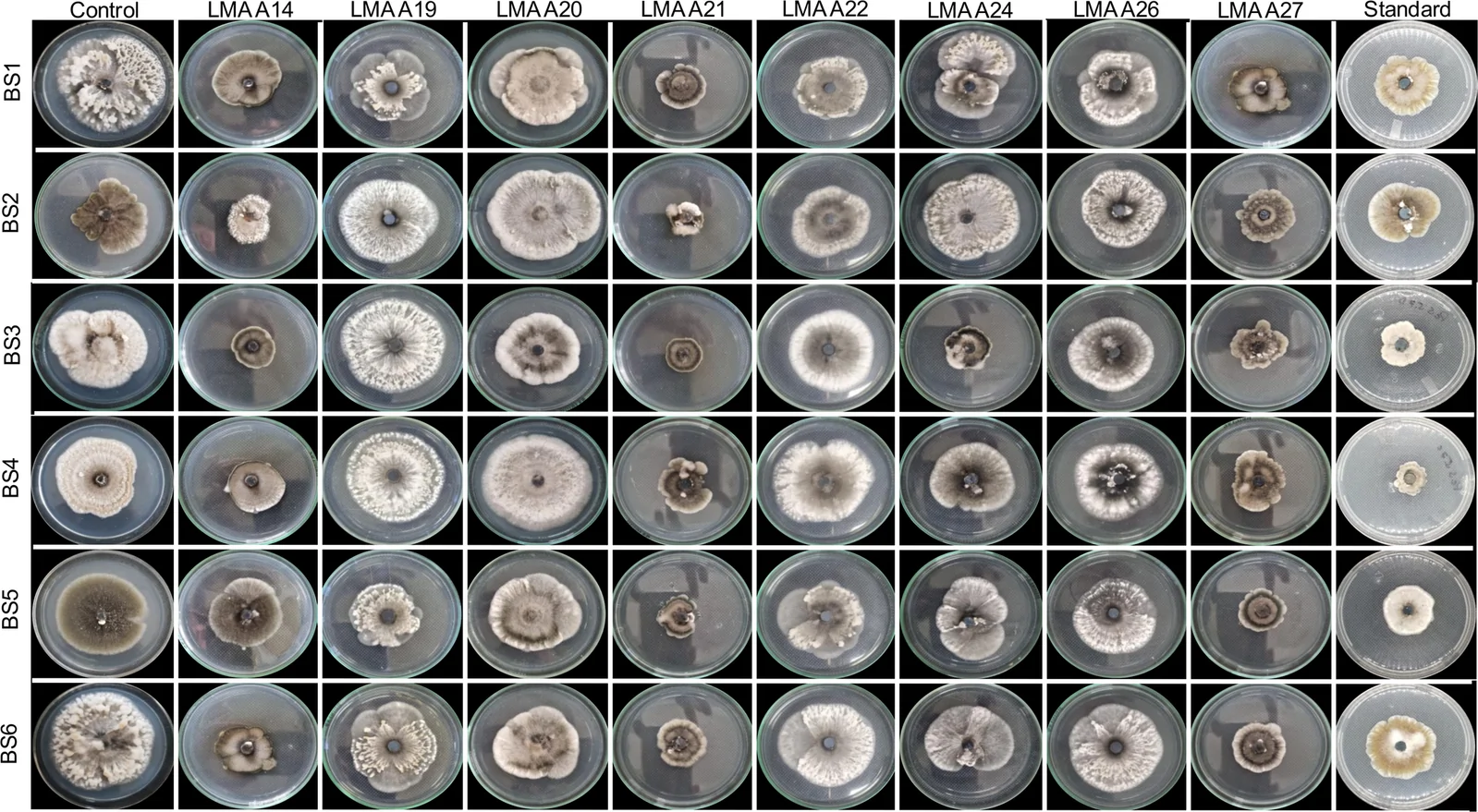
Inhibition of *B. sorokiniana* by non-volatile secondary metabolites (non-VOCs) produced by *Trichoderma* isolates. Cell-free culture filtrates were incorporated into PDA at 25% (v/v). For the control treatment, plates consisted of PDA medium mixed with sterile uninoculated broth instead of the fungal filtrate. Plates were incubated at 25 ± 2 °C for seven days

Among the native isolates, *T. longibrachiatum* LMA A21 was the most effective, inhibiting all six pathogen isolates with inhibition values ranging from 50.17% to 74.77%. *T. harzianum* LMA A14, *T. asperelloides* LMA A20, *T. virens* LMA A27, and the commercial standard inhibited only two pathogen isolates each (Fig. 5).

Inhibition profiles varied markedly among pathogen isolates. BS6 was the least susceptible, showing inhibition values from − 38.35 to 50.17% and six of the antagonists failed to significantly inhibit its growth (Table 4, Table S4). In contrast, BS3 was the most susceptible, exhibiting inhibition levels ranging from 19.66 to 68.91% when exposed to all eight antagonists.

## Discussion

In the present study, eight local *Trichoderma* isolates were evaluated for their antifungal potential against different isolates of the phytopathogen *B. sorokiniana*. Although the ITS region is the official primary barcode for fungi, species-level resolution within the genus *Bipolaris* can sometimes be limited when relying on this marker alone. To overcome this limitation and provide a robust framework for our biocontrol assays, a polyphasic approach was adopted. The morphological and cultural characteristics of the target isolates were evaluated together with molecular sequence data and phylogenetic inference, while pathogenicity was assessed through artificial inoculation of barley plants. The combined evidence supported the identification of the isolates as *B. sorokiniana* and confirmed their pathogenicity to barley.

Overall, all *Trichoderma* isolates suppressed pathogen growth in vitro, reinforcing the value of native strains adapted to local conditions as potential biocontrol agents. In dual-culture assays, all isolates displayed antagonistic activity, although with variable efficiency. Such variability is expected, since the expression of key antagonistic traits, including enzyme production, secondary metabolite profiles, and competitive colonization, may differ substantially among *Trichoderma* isolates. Similar isolate-specific responses were reported by Rodríguez-Martínez et al. (2025) when evaluating antagonism against *Rhizoctonia solani*, *Phytophthora capsici*, and *Fusarium* spp., highlighting the importance of screening multiple candidates when identifying effective biocontrol agents. Our data also indicate that this interaction is not unidirectional: the antagonistic response varied among *B. sorokiniana* isolates, and the efficiency of each *Trichoderma* isolate differed depending on the pathogen isolate encountered.

In addition to the genetic backgrounds of both antagonist and pathogen, the physiological traits of *Trichoderma* isolates may also have contributed to the observed differences in antagonistic performance. Variations in mycelial growth rate, resource acquisition, and the production and diffusion of antifungal metabolites have been reported to affect the outcome of fungal interactions in vitro (Kubiak et al. 2023; Hernández et al. 2024).

Similarly, isolates with faster colony expansion may occupy the available substrate more rapidly, restricting pathogen growth before extensive metabolite-mediated inhibition occurs (Nguyen et al. 2025). Therefore, the antagonistic profiles observed here may reflect the combined influence of genetic and physiological traits, as well as the complex interactions between the two organisms.

Among the isolates evaluated, *T. harzianum* LMA A14, *T. asperelloides* LMA A20, and *T. azevedoi* LMA A22 showed the strongest inhibition in dual culture, followed by *T. asperelloides* LMA A24. These findings are consistent with previous reports demonstrating the high antagonistic potential of *T. harzianum*, including 90.12% inhibition of *Fusarium oxysporum* (Girma 2022) and inhibition rates above 76% and 90% against *B. sorokiniana* (Erdurmuş and Katırcıoğlu 2009; Villa-Rodríguez et al. 2022). Likewise, studies have shown that *T. harzianum* can achieve up to 86% inhibition of *B. sorokiniana* (Raj et al. 2024), supporting the robust performance observed for LMA A14 in our assay.

Intraspecific variation in phytopathogenic fungi can lead to differences in cell wall composition, toxin production, and enzymatic detoxification pathways capable of neutralizing *Trichoderma* secondary metabolites (Apoga et al. 2002; Ghazvini and Tekauz 2007; Gow et al. 2017). Consequently, a single *Trichoderma* isolate, such as LMA A14, may encounter different levels of physiological resistance depending on the pathogen strain. This bidirectional, isolate-dependent interaction explains why standardizing biocontrol evaluations against a single reference strain may overestimate or underestimate an antagonist’s true field potential, underscoring the ecological complexity of these multi-genotype fungal interactions.

Several findings support the biocontrol patterns observed here. For instance, *T. azevedoi* and *T. asperelloides* strains have been documented as effective antagonists against a broad spectrum of phytopathogens, with inhibition rates matching or exceeding those reported in previous in vitro assessments (Silva et al. 2022; Ruangwong et al. 2021).

In our dual-culture assays, the observed antagonism suggests a potential combination of antibiosis and aggressive mycoparasitism as the underlying mechanisms. Most *B. sorokiniana* colonies showed macroscopic features consistent with mycoparasitic behavior, such as overgrowth and extensive sporulation by isolates LMA A20, LMA A14, LMA A19, LMA A22, LMA A24, and LMA A26 (El-Komy et al. 2022).

The structural degradation and the efficiency of mycoparasitism are known to vary significantly among *Trichoderma* species (Wang and Zhuang 2019; Mukherjee et al. 2022). The process involves hyphal penetration mediated by the secretion of cell wall-degrading enzymes, particularly chitinases and β-1,3-glucanases (Xue et al. 2021; Singh et al. 2024). In this study, the rapid colonization and physical displacement of *B. sorokiniana* further support the crucial role of these hydrolytic pathways in the competitive success of native antagonists.

Genomic heterogeneity among *Trichoderma* species, particularly in the repertoire of genes encoding hydrolytic enzymes and secondary metabolites, may help explain the distinct and superior antagonistic levels observed for isolates LMA A14 and LMA A20 (Alfiky and Weisskopf 2021). Antibiosis may also be involved in suppressing *B. sorokiniana*. Clear inhibition zones lacking pathogen hyphae were produced by *T. virens* LMA A27, *T. longibrachiatum* LMA A21, and *T. lentiforme* LMA A26, a phenotype potentially associated with secretion of bioactive compounds such as peptaibols, polyketides, terpenes, and pyrones (Hou et al. 2021; Ma et al. 2023; Nguyen et al. 2025).

Interestingly, some *Trichoderma* isolates expressed multiple antagonistic behaviors depending on the target pathogen isolate. For example, *T. lentiforme* LMA A26 combined rapid colony expansion, inhibition-zone formation, and potential mycoparasitism behavior, reinforcing that *Trichoderma* spp. frequently employ complementary mechanisms that act simultaneously against phytopathogens (Paul et al. 2021; Poletto et al. 2024).

The volatile organic compounds (VOCs) released by *Trichoderma* isolates inhibited *B. sorokiniana* growth to varying degrees, reflecting the extensive metabolic diversity associated with volatile production in this genus. Rather than acting as universal inhibitors, VOC-mediated activity is highly isolate-specific and depends on unique chemical profiles (van Zijl de Jong et al. 2023). This inhibition is further modulated by bidirectional chemical cross-talk, as volatile compounds produced by the pathogen itself can interact with and influence *Trichoderma* metabolites (Momtaz et al. 2022). Functionally, the synthesis of these diverse volatile mixtures provides an ecological advantage by suppressing competitors, increasing resource availability, and facilitating rapid substrate colonization (Fishal et al. 2022; Abdenaceur et al. 2024).

In this work, *T. asperelloides* LMA A20 and *T. longibrachiatum* LMA A21 were the most effective volatile producers against *B. sorokiniana*. This performance is consistent with previous literature documenting the strong volatile activity of both *T. asperelloides* (Heflish et al. 2021) and *T. longibrachiatum* (Joo and Hussein 2022) against broad-spectrum phytopathogens. Taken together, these findings indicate that the volatile antagonistic potential observed in LMA A20 and LMA A21 is a conserved and effective mechanism for suppressing fungal targets.

Among the antagonists evaluated, *T. longibrachiatum* LMA A21 was the most effective in suppressing *B. sorokiniana* growth through non-volatile metabolite production, a phenotype typical of inhibition mediated by highly diffusible bioactive compounds (Adnan et al. 2019; Sawant 2014; Stange et al. 2023). Conversely, *T. asperelloides* LMA A20 displayed limited activity in this specific assay, suggesting that its antagonistic profile relies more heavily on alternative mechanisms, such as VOC production or aggressive mycoparasitism (Go et al. 2023). This metabolic variability aligns with Sazonova et al. (2025), who noted that the capacity to secrete appreciable levels of non-volatile antifungal metabolites is not universally conserved among all *Trichoderma* strains. The pronounced suppressive effects of *T. longibrachiatum* cell-free culture filtrates observed here are further supported by literature showing their fungistatic mode of action, which induces hyphal abnormalities and restricts mycelial expansion across diverse plant pathogens (Sridharan et al. 2020).

It must also be considered that the inhibitory effect exerted by *Trichoderma* cell-free culture filtrates is driven by a combination of non-volatile factors. Rather than being solely restricted to specialized secondary metabolites, this mechanism is also influenced by the release of primary metabolites and physicochemical alterations in the environment, such as changes in pH and nutrient depletion during prior cultivation. However, the pH of the culture filtrate was not measured in the present study, which precludes a direct assessment of its individual contribution to the observed inhibitory effect. Future studies should include filtrate pH measurements and pH-adjusted controls to disentangle this factor from the activity of secondary metabolites.

Furthermore, isolates belonging to the same *Trichoderma* species differed markedly in their antagonistic profiles (Izidoro-Morimitsu et al. 2026). Although *T. asperelloides* LMA A20 and LMA A24 displayed similar mechanisms, the two *T. harzianum* isolates differed substantially in volatile and non-volatile metabolite production, highlighting the pronounced intraspecific variability commonly observed within the genus.

The commercial *T. harzianum* product used as a standard treatment exhibited high inhibition levels in vitro, reinforcing the well-documented capacity of this species to suppress a wide range of phytopathogens (Swathy et al. 2024). This consistent performance across studies reflects the extensive validation *T. harzianum* has undergone for biocontrol applications, aligning with the inhibitory activity observed in the present work. Nevertheless, although dual-culture and metabolite assays provide robust evidence of antagonism, future studies incorporating specific enzymatic assays, advanced microscopy, and metabolomic characterization via GC-MS or LC-MS remain necessary to identify and quantify the precise physical and chemical mechanisms involved.

Our in vitro screening revealed that *B. sorokiniana* isolates showed distinct levels of susceptibility to the same *Trichoderma* antagonist, with certain pathogen isolates consistently exhibiting low inhibition across multiple mechanisms. For instance, isolate BS4 was minimally affected by volatile metabolites, demonstrating that antifungal efficacy depends on the intrinsic characteristics of the target pathogen. This variable susceptibility may reflect differences in the physiological and biochemical characteristics of *B. sorokiniana* isolates, potentially including variation in enzymatic profiles and detoxification mechanisms (Al-Sadi 2016).

Studies conducted worldwide indicate that *B. sorokiniana* isolates from barley and wheat exhibit intense genetic heterogeneity, accompanied by marked variation in morphology, pathogenicity, and virulence (Ghazvini and Tekauz 2008; Persson et al. 2009; Yadav et al. 2013; Verma et al. 2020). This high genetic variability is consistent with the phenotypic and molecular diversity observed here, where macromorphological traits occasionally fell outside established cultural groups (Gupta et al. 2018; Yadav et al. 2022). Our findings agree with Mann et al. (2014), who reported extensive morphological variability in monoconidial isolates, reinforcing the highly heterogeneous nature of the studied isolates.

Consistently, our ISSR analysis confirmed this high genetic variability, evidenced by a low average similarity coefficient (0.30) and supported by relatively high values of Nei’s genetic diversity (*h* = 0.444) and Shannon’s information index (*I* = 0.634). These metrics indicate a highly polymorphic genetic structure, and the UPGMA clustering did not reflect their geographic origin. Notably, the dendrogram revealed that morphological resemblance does not mirror genetic relatedness; for instance, isolates BS1 and BS6 shared similar macromorphological traits but were assigned to distinct clades. Furthermore, the co-clustering of isolates from Guarapuava and Ponta Grossa suggests the absence of a strict geographic pattern regarding the genetic distribution of the studied isolates.

This observed distribution may be attributed to the high dispersal capacity of *B. sorokiniana* via infected seeds, anemochory, and crop residues, which facilitates pathogen movement and maintains high genetic diversity across geographic regions. Furthermore, since the teleomorph (sexual stage) of this pathogen is rarely observed under natural conditions (Roy et al. 2023), this elevated genetic variability is likely sustained by a combination of mutation, parasexual recombination, and demographic factors (Sharma et al. 2022). Such multi-genotypic diversity has important implications for disease management, as shifting pathogen populations may display different levels of aggressiveness and unpredictable responses to control strategies.

In this context, the separation of isolate BS4 as a separate branch and isolate BS6 into a distinct clade, despite their shared low susceptibility to certain *Trichoderma* metabolites, is noteworthy. This pattern indicates that isolates with different genetic profiles may exhibit similar levels of susceptibility to specific antagonistic metabolites, suggesting that isolate-dependent responses are not necessarily associated with overall genetic relatedness. It also reinforces the need for future screening programs to include diverse pathogen isolates from different genetic backgrounds, so that robust *Trichoderma* strains can be selected against the variable susceptibility patterns observed among different pathogen isolates.

Furthermore, information on the genetic variability of *B. sorokiniana* in Brazil remains limited, as existing studies have focused mainly on wheat-derived isolates (Müller and Germani 2005; Mann et al. 2014). Consequently, the diversity of populations infecting barley in the country remains largely unexplored. Advancing this knowledge is essential to ensure the consistency of disease-management strategies, particularly given the observed variation in susceptibility to biocontrol agents among pathogen isolates. However, considering our regional sample size, these genetic patterns should be treated as preliminary; larger population-level samplings are required to characterize genetic structure and regional gene flow more comprehensively.

Finally, although our in vitro assays provide strong screening evidence, they do not replace validation under greenhouse or field conditions, where environmental fluctuations, soil-microbe interactions, and host physiological responses can strongly influence biocontrol performance. Future research should therefore move toward controlled-environment and multi-location field trials to evaluate application methods, assess compatibility with the native microbiota, and determine the long-term stability and practical applicability of these native *Trichoderma* isolates.

## Conclusions

The present study demonstrated that native *Trichoderma* isolates differ markedly in their ability to suppress genetically diverse *B. sorokiniana* isolates in vitro, with antagonistic efficacy varying among pathogen isolates. ISSR analysis revealed high genetic diversity among *B. sorokiniana* isolates, with no obvious relationship between ISSR clustering and sampling location observed among the six isolates examined, supporting the inclusion of genetically diverse isolates in future biocontrol screening programs.

The significant interaction between *Trichoderma* and *B. sorokiniana* across all antagonism assays demonstrated that the antagonistic response varied among pathogen isolates. Among the *Trichoderma* strains evaluated, *T. harzianum* LMA A14, *T. azevedoi* LMA A22, *T. asperelloides* LMA A20 and LMA A24, and *T. longibrachiatum* LMA A21 showed the strongest antagonistic performance, albeit through different and complementary antagonistic behaviors. These findings suggest that screening antagonists against a single pathogen isolate may underestimate or overestimate their true biocontrol potential.

Overall, these results provide a solid basis for developing sustainable strategies to manage barley spot blotch. However, greenhouse and multilocation field trials are still needed to validate the stability and consistency of these antagonists under natural conditions. In addition, the microbial diversity of threatened ecosystems such as the Araucaria Forest may represent an important source of regionally adapted *Trichoderma* strains with broad antagonistic potential, strengthening sustainable disease management and improving the resilience of barley production systems.

## Supplementary Information

Below is the link to the electronic supplementary material.

Supplementary Material 1

Supplementary Material 2

Supplementary Material 3

Supplementary Material 4

Supplementary Material 5

## Data Availability

The datasets generated and analyzed during the current study are available from the corresponding author on reasonable request.
