# Supplementary material for: Antagonistic responses of native Trichoderma spp. vary among Bipolaris sorokiniana isolates
Source: Arch Microbiol. 2026 Sep 26;208(12):662. doi: 10.1007/s00203-026-05225-6 (PMC13615924; doi:10.1007/s00203-026-05225-6)
Supplement: Supplementary file 1 [file 203_2026_5225_MOESM2_ESM.docx]

Table S1 GenBank accession numbers for the ribosomal (ITS) and protein-coding (*tef1*-α and *rpb2*) gene sequences of the *Trichoderma* isolates

| Isolate | Species | GenBank accession | | |
| --- | --- | --- | --- | --- |
|  |  | ITS | *tef1-α* | *rpb2* |
| LMA A14 | *T. harzianum* | PX590048 | PX578866 | - |
| LMA A19 | *T. harzianum* | PX590049 | PX578889 | - |
| LMA A20 | *T. asperelloides* | PX590046 | PX578885 | - |
| LMA A24 | *T. asperelloides* | PX590064 | PX578872 | PX578910 |
| LMA A21 | *T. longibrachiatum* | PX590074 | PX578876 | PX578911 |
| LMA A22 | *T. azevedoi* | PX590053 | PX578892 | PX578901 |
| LMA A26 | *T. lentiforme* | PX590058 | PX578896 | PX578902 |
| LMA A27 | *T. virens* | PX590044 | PX578865 | PX578900 |
