# Supplementary material for: Antagonistic responses of native Trichoderma spp. vary among Bipolaris sorokiniana isolates
Source: Arch Microbiol. 2026 Sep 26;208(12):662. doi: 10.1007/s00203-026-05225-6 (PMC13615924; doi:10.1007/s00203-026-05225-6)
Supplement: Supplementary file 2 [file 203_2026_5225_MOESM3_ESM.docx]

Table 2 *In vitro* mycelial growth of *Bipolaris sorokiniana* isolates in dual culture with *Trichoderma* isolates after seven days at 25 ± 2 °C. Values represent mean colony diameter (cm) ± standard deviation.

| ***Trichoderma* isolates** | **Mycelial growth (cm)** | | | | | | | |  |
| --- | --- | --- | --- | --- | --- | --- | --- | --- | --- |
|  | ***B. sorokiniana* isolates** | | | | | | | |  |
|  | **BS1** | **BS2** | **BS3** | **BS4** | **BS5** | | **BS6** | | |
| **Control** | 5.30 ± 0.27 | 5.70 ± 0.18^*^ | 5.20 ± 0.15 | 5.53 ± 0.36 | | 5.2 ± 0,54 | | 5.43 ± 0.36 |  |
| ***T. harzianum* LMA A14** | 1.17 ± 0.32^*^ | 1.01 ± 0.05^*^ | 1.37 ± 0.17^*^ | 1.33 ± 0.13^*^ | | 1.79 ± 0.13^*^ | | 1.48 ± 0.26^*^ |  |
| ***T. harzianum* LMA A19** | 1.08 ± 0.18^*^ | 1.64 ± 0.06^*^ | 1.48 ± 0.05^*^ | 1.68 ± 0.02^*^ | | 1.32 ± 0.30^*^ | | 1.77 ± 0.06^*^ |  |
| ***T. asperelloides* LMA A20** | 1.32 ± 0.40^*^ | 1.94 ± 0.20^*^ | 1.67 ± 0.16^*^ | 1.63 ± 0.03^*^ | | 1.44 ± 0.08^*^ | | 1.10 ± 0.42^*^ |  |
| ***T. longibrachiatum* LMA A21** | 1.78 ± 0.38^*^ | 2.12 ± 0.26^*^ | 2.5 ± 0.17^*^ | 2.33 ± 0.06^*^ | | 2.22 ± 0.17^*^ | | 1.89 ± 0.13^*^ |  |
| ***T. azevedoi* LMA A22** | 1.02 ± 0.01^*^ | 1.50 ± 0.14^*^ | 1.23 ± 0.19^*^ | 1.36 ± 0.01^*^ | | 1.14 ± 0.28^*^ | | 1.40 ± 0.14^*^ |  |
| ***T. asperelloides* LMA A24** | 1.19 ± 0.15^*^ | 1.19 ± 0.41^*^ | 0.92 ± 0.23^*^ | 1.69 ± 0.16^*^ | | 1.41 ± 0.10^*^ | | 1.49 ± 0.30^*^ |  |
| ***T. lentiforme* LMA A26** | 2.07 ± 0,19^*^ | 2.13 ± 0.29^*^ | 2.17 ± 0.26^*^ | 1.80 ± 0.09^*^ | | 1.37 ± 0.37^*^ | | 1.83 ± 0.27^*^ |  |
| ***T. virens* LMA A27** | 2.12 ± 0.04^*^ | 1.80 ± 0.37^*^ | 2.19 ± 0.25^*^ | 1.72 ± 0.02^*^ | | 1.91 ± 0.34^*^ | | 1.89 ± 0.26^*^ |  |
| **Standard**  **(*T. harzianum)*** | 0.76 ± 0.08^*^ | 0.50 ± 0.10^*^ | 0.29 ± 0.23^*^ | 1.00 ± 0.06^*^ | | 0.87 ± 0.12^*^ | | 0.98 ± 0.15^*^ |  |

^*^ Indicates a significant difference between the *Trichoderma* treatment and the pathogen-only control according to Dunnett’s many-to-one test (p < 0.001). Values represent the mean of three pooled observations (N = 3), with each pooled observation calculated as the mean of the corresponding replicate from the two independent experimental runs.
