# Supplementary material for: Antagonistic responses of native Trichoderma spp. vary among Bipolaris sorokiniana isolates
Source: Arch Microbiol. 2026 Sep 26;208(12):662. doi: 10.1007/s00203-026-05225-6 (PMC13615924; doi:10.1007/s00203-026-05225-6)
Supplement: Supplementary file 3 [file 203_2026_5225_MOESM4_ESM.docx]

Table 3 *In vitro* mycelial growth of *B. sorokiniana* under the influence of volatile secondary metabolites (VOCs) produced by *Trichoderma* isolates. Plates were incubated at 25 ± 2 °C for seven days. Values represent mean colony diameter (cm) ± standard deviation.

| ***Trichoderma* isolates** | | **Mycelial growth (cm)** | | | | | | | | | |  |
| --- | --- | --- | --- | --- | --- | --- | --- | --- | --- | --- | --- | --- |
|  |  | ***B. sorokiniana* isolates** | | | | | | | | | |  |
|  |  | **BS1** | **BS2** | **BS3** | | **BS4** | | **BS5** | | **BS6** | |  |
| **Control** | 5.62 ± 0.52 | | 7.08 ± 0.74 | | 6.74 ± 0.99 | | 6.73 ± 2.32 | | 6.68 ± 0.21 | | 3.09 ± 0.60 | |
| ***T. harzianum* LMA A14** | 3.20 ± 0.38 ^*^ | | 6.11 ± 0.83^*^ | | 4.83 ± 0.71^*^ | | 6.92 ± 1.06 | | 3.29 ± 0.50^*^ | | 5.00 ± 0.27^**^ | |
| ***T. harzianum* LMA A19** | 2.24 ± 0.08 ^*^ | | 4.18 ± 0.35^*^ | | 3.29 ± 0.57^*^ | | 2.76 ± 0.20^*^ | | 3.94 ± 0.48^*^ | | 4.13 ± 0.28^**^ | |
| ***T. asperelloides* LM A20** | 2.75 ± 0.25^*^ | | 3.33 ± 0.52^*^ | | 3.20 ± 0.78^*^ | | 3.54 ± 0.45^*^ | | 2.32 ± 0.24^*^ | | 1.81 ± 0.26^*^ | |
| ***T. longibrachiatum* LMA A21** | 2.96 ± 0.43 ^*^ | | 4.98 ± 0.58^*^ | | 4.10 ± 0.32^*^ | | 4.45 ± 0.57^*^ | | 4.01 ± 0.49^*^ | | 5.82 ± 0.94^**^ | |
| ***T. azevedoi* LMA A22** | 4.41 ± 0.83 ^*^ | | 5.16 ± 0.47^*^ | | 3.77 ± 0.04^*^ | | 3.70 ± 0.49^*^ | | 2.44 ± 0.26^*^ | | 2.58 ± 0.55^*^ | |
| ***T. asperelloides* LMA A24** | 2.21 ± 0.36^*^ | | 4.20 ± 0.59^*^ | | 2.22 ± 0.06^*^ | | 3.65 ± 0.34^*^ | | 3.31 ± 0.58^*^ | | 2.07 ± 0.42^*^ | |
| ***T. lentiforme* LMA A26** | 3.4 ± 0.40^*^ | | 3.22 ± 0.28^*^ | | 2.54 ± 0.08^*^ | | 4.71 ± 0.48^*^ | | 4.00 ± 0.23^*^ | | 2.62 ± 0.10^*^ | |
| ***T. virens* LMA A27** | 4.23 ± 0.62^*^ | | 3.02 ± 0.64^*^ | | 3.00 ± 0.52^*^ | | 5.62 ± 0.51 | | 2.92 ± 0.46^*^ | | 2.44 ± 0.38^*^ | |
| **Standard (*T. harzianum)*** | 2.17 ± 0.14^*^ | | 1.45 ± 0.17^*^ | | 3.04 ± 0.09^*^ | | 2.59 ± 0.13^*^ | | 3.32 ± 0.34^*^ | | 1.38 ± 0.08^*^ | |

^*^Indicates a significant difference between the *Trichoderma* treatment and the pathogen-only control according to Dunnett’s many-to-one test (p < 0.05). A single asterisk (^*^) indicates significantly lower pathogen growth than the pathogen-only control, whereas two asterisks (^**^) indicate significantly higher pathogen growth than the pathogen-only control. Values represent the mean of three pooled observations (N = 3), with each pooled observation calculated as the mean of the corresponding replicate from the two independent experimental runs.
