# Supplementary material for: Antagonistic responses of native Trichoderma spp. vary among Bipolaris sorokiniana isolates
Source: Arch Microbiol. 2026 Sep 26;208(12):662. doi: 10.1007/s00203-026-05225-6 (PMC13615924; doi:10.1007/s00203-026-05225-6)
Supplement: Supplementary file 4 [file 203_2026_5225_MOESM5_ESM.docx]

Table 4 Effect of cell-free culture filtrates from *Trichoderma* isolates on the mycelial growth of *B. sorokiniana*. Plates were incubated at 25 ± 2 °C for seven days. Values represent mean colony diameter (cm) ± standard deviation.

| ***Trichoderma* isolates** | **Mycelial growth (cm)** | | | | | |  |
| --- | --- | --- | --- | --- | --- | --- | --- |
|  | ***B. sorokiniana* isolates** | | | | | |  |
|  | **BS1** | **BS2** | **BS3** | **BS4** | **BS5** | **BS6** |  |
| **Control** | 7.55 ± 1.02 | 8.05 ± 0.27 | 7.14 ± 0.36 | 7.24 ± 0.18 | 6.68 ± 0.21 | 4.88 ± 1.45 | |
| ***T. harzianum* LMA A14** | 4.82 ± 0.56^*^ | 3.24 ± 0.42^*^ | 3.20 ± 0.27^*^ | 4.44 ± 0.51^*^ | 3.90 ± 0.19^*^ | 3.75 ± 0.45 | |
| ***T. harzianum* LMA A19** | 6.38 ± 0.43^*^ | 6.76 ± 0.60^*^ | 4.02 ± 0.87^*^ | 5.28 ± 0.59^*^ | 5.21 ± 0.16^*^ | 5.96 ± 0.92 | |
| ***T. asperelloides* LMA A20** | 7.19 ± 0.35 | 5.84 ± 0.78^*^ | 5.74 ± 0.48^*^ | 7.76 ± 0.25 | 6.25 ± 0.39 | 6.75 ± 0.55^**^ | |
| ***T. longibrachiatum* LMA A21** | 2.57 ± 0.10^*^ | 2.03 ± 0.13^*^ | 2.22 ± 0.14^*^ | 1.90 ± 0.02^*^ | 2.45 ± 0.13^*^ | 2.43 ± 0.05^*^ | |
| ***T. azevedoi* LMA A22** | 5.04 ± 0.69^*^ | 5.39 ± 0.35^*^ | 5.48 ± 0.59^*^ | 6.16 ± 0.53^*^ | 5.02 ± 0.86^*^ | 6.05 ± 0.45 | |
| ***T. asperelloides* LMA A24** | 5.30 ± 0.94^*^ | 6.05 ± 0.57^*^ | 3.87 ± 0.74^*^ | 6.01 ± 0.47^*^ | 5.26 ± 0.44^*^ | 5.90 ± 0.36 | |
| ***T. lentiforme* LMA A26** | 6.31 ± 0.28^*^ | 5.71 ± 0.59^*^ | 5.54 ± 0.85^*^ | 6.10 ± 0.11^*^ | 5.59 ± 0.81^*^ | 6.38 ± 0.13^**^ | |
| ***T. virens* LMA A27** | 3.49 ± 0.09^*^ | 3.81 ± 0.59^*^ | 3.61 ± 0.53^*^ | 4.59 ± 0.18^*^ | 3.39 ± 0.10^*^ | 3.82 ± 0.14 | |
| **Standard (*T. harzianum)*** | 4.33 ± 0.11^*^ | 4.6 ± 0.47^*^ | 3.02 ± 0.23^*^ | 2.45 ± 0.18^*^ | 3.78 ± 0.36^*^ | 4.47 ± 0.00 | |

^*^ Indicates a significant difference between the *Trichoderma* treatment and the pathogen-only control according to Dunnett’s many-to-one test (p < 0.05). A single asterisk (^*^) indicates significantly lower pathogen growth than the pathogen-only control, whereas two asterisks (^**^) indicate significantly higher pathogen growth than the pathogen-only control. Values represent the mean of three pooled observations (N = 3), with each pooled observation calculated as the mean of the corresponding replicate from the two independent experimental runs.
